# Telomere Length Measurement by Molecular Combing

**DOI:** 10.3389/fcell.2020.00493

**Published:** 2020-06-16

**Authors:** Vivian F. S. Kahl, Joshua A. M. Allen, Christopher B. Nelson, Alexander P. Sobinoff, Michael Lee, Tatjana Kilo, Raja S. Vasireddy, Hilda A. Pickett

**Affiliations:** ^1^Telomere Length Regulation Unit, Children’s Medical Research Institute, Faculty of Medicine and Health, The University of Sydney, Sydney, NSW, Australia; ^2^Department of Hematology, Children’s Hospital at Westmead, Sydney Children’s Hospitals Network, Sydney, NSW, Australia

**Keywords:** telomere, telomere length, senescence, Telomere length Combing Assay, telomerase

## Abstract

Telomeres are repetitive regions of DNA bound by specialized proteins at the termini of linear chromosomes that prevent the natural chromosome ends from being recognized as DNA double strand breaks. Telomeric DNA is gradually eroded with each round of cell division, resulting in the accumulation of critically short or dysfunctional telomeres that eventually trigger cellular senescence. Consequently, telomere length is indicative of the proliferative capacity of a cell. Multiple methods exist to measure telomere length and telomere content, but a simple and reliable technique to accurately measure individual telomere lengths is currently lacking. We have developed the Telomere length Combing Assay (TCA) to measure telomere length on stretched DNA fibers. We used TCA to measure telomere erosion in primary human fibroblasts, and to detect telomere lengthening in response to activation of telomere maintenance pathways. TCA was also used to accurately measure telomere length in healthy individuals, and to identify critically short telomeres in patients with telomere biology disorders. TCA is performed on isolated DNA, negating the need for cycling cells. TCA is amenable to semi-automated image analysis, and can be fully automated using the Genomic Vision molecular combing platform. This not only precludes sampling bias, but also provides the potential for high-throughput applications and clinical development. TCA is a simple and versatile technique to measure the distribution of individual telomere lengths in a cell population, offering improved accuracy, and more detailed biological insight for telomere length measurement applications.

## Introduction

Telomeres are specialized nucleoprotein structures at the ends of linear chromosomes that function to protect the chromosome ends, thereby maintaining the stability of the genome. Telomeric DNA comprises repetitive sequences of the hexanucleotide TTAGGG_n_ repeat unit, bound in a sequence-specific manner to the protein complex shelterin, and assembled into macromolecular structures called telomere-loops (t-loops; Griffith et al., 1999; Blackburn, 2000; Van Ly et al., 2018). In normal human somatic cells, telomeres range from 5–15 kb in length (Pickett et al., 2011), and telomere length variability exists between individual telomeres and between different cell types. Inter-individual variability is also observed across the human population, superimposed on the well-established age-associated decline in telomere length (Aubert and Lansdorp, 2008). The negative correlation between telomere length and chronological age is attributed to terminal replication limitations, oxidative damage, and nucleolytic degradation (Harley et al., 1990; Baird, 2008a).

The telomere attrition that accompanies cellular proliferation eventually leads to an accumulation of critically short or unprotected telomeres, which signals the onset of replicative senescence (Hayflick and Moorhead, 1961; Kaul et al., 2012). Senescence provides a barrier to unlimited cellular proliferation, thereby fulfilling a potent tumor suppressive role (Reddel, 2010). In some rare cases, cells are able to bypass replicative senescence by inactivating tumor suppressor pathways, allowing cells to proceed into crisis, which is characterized by catastrophic telomere shortening and widespread genome instability (Reddel, 2010). This process provokes the emergence of cancer cells with tumorigenic advantage. However, oncogenic progression necessitates stabilization of the genome to overcome crisis, which is dependent upon activation of a telomere maintenance mechanism (TMM; Maciejewski and de Lange, 2017). Telomere maintenance is achieved by one of two defined mechanisms. First, activation of the ribonucleoprotein enzyme telomerase, which uses a template sequence embedded within the RNA component of the enzyme to reverse transcribe telomeric sequences directly onto the chromosome termini (Bryan et al., 1998; Reddel, 2014). Second, the Alternative Lengthening of Telomeres (ALT) pathway, which co-opts homology-directed repair mechanisms to drive template-mediated telomere extension (Cesare and Reddel, 2010; Reddel, 2014; Pickett and Reddel, 2015).

Telomerase is also active in the germline, during embryogenesis, and in hematopoietic, stem and rapidly renewing cells, but is suppressed to undetectable levels upon differentiation in human somatic cells. In this capacity, telomerase supports the proliferative requirements of the organism by maintaining telomere length. When telomere length cannot be adequately maintained, highly proliferative tissues become impacted. Telomere biology disorders (TBDs) are a group of diseases caused by germline mutations in genes involved in telomere maintenance and function (Bertuch, 2016). The clinical manifestations of these disorders include bone marrow failure, aplastic anemia, pulmonary fibrosis and acute myeloid leukemia, which are attributed to the premature loss of stem cell populations. TBDs are typically characterized by telomere lengths in the bottom percentile of the normal population, and telomere length measurement is used for the clinical diagnosis of patients, bone marrow donor screening, and to direct effective treatment regimens for bone marrow transplant. Milder deficiencies in telomere length have also been implicated as risk factors in several diseases, including cardiovascular disease, diabetes mellitus, obesity, liver cirrhosis, and cancer (Epel et al., 2004; Sampson and Hughes, 2006; Willeit et al., 2010; Haycock et al., 2014; Opresko and Shay, 2016).

A variety of telomere length measurement methods exist, each with its own strengths and weaknesses. Accuracy and precision are critical for the utility of telomere length measurement in clinical, epidemiological and research studies. Many current measurement techniques provide an average or relative measurement of telomere length or telomere content; however, it is well established that it is the shortest telomeres, rather than average telomere length, that trigger cellular senescence (Hemann et al., 2001; Herbig et al., 2004; Zou et al., 2004; Kaul et al., 2012). Measurement techniques that provide the distribution of telomere lengths in a cell population offer improved and more informative data regarding telomere length dynamics.

Here, we have developed the Telomere length Combing Assay (TCA) as an accurate and robust technique to measure the distribution of telomere lengths in a cell population. TCA involves stretching DNA fibers onto coated glass coverslips using a constant stretching factor (Schurra and Bensimon, 2009). Telomeric DNA is then visualized with telomere-specific PNA probes, and individual telomere lengths are measured manually or using automated software and converted to absolute telomere length measurements according to the stretching factor. We demonstrate that telomere length measurements obtained by TCA are comparable to other widely utilized methods, and that TCA can detect dynamic changes in telomere length. TCA can be used to measure telomere lengths in healthy individuals and can accurately identify TBD patients with critically short telomeres. Finally, we demonstrate that TCA is amenable to semi-automated image analysis through two open-source software platforms and fully automated image analysis using the Genomic Vision molecular combing platform, and can therefore be adapted for high-throughput applications.

## Materials and Methods

### Cell Culture

The cell lines U-2 OS, HeLa, HT1080, HT1080 hTR, and IIICF/c were cultured in Dulbecco’s Modified Eagle’s Medium (DMEM; Gibco, Paisley, United Kingdom) with 10% fetal bovine serum (FBS) at 37°C in 10% CO_2_. MRC-5 cells were cultured similarly, but in 5% CO_2_. The CCRF-CEM cell line was cultured in RPMI (Thermo Scientific, Norwood, Australia) with 10% FBS. All cell lines were authenticated by 16-locus short-tandem-repeat profiling and confirmed to be free of mycoplasma contamination by CellBank Australia (Children’s Medical Research Institute, Westmead, Australia).

### Subjects

Peripheral blood was collected from healthy individuals and clinically referred individuals suspected of having a TBD through the Department of Hematology Telomere Length Testing Facility, Sydney Children’s Hospitals Network. Informed consent was obtained from all participating individuals, and the studies were approved by the Human Research Ethics Committee of the Sydney Children’s Hospitals Network.

### Genomic DNA Extraction

Cells were harvested with 0.05% Trypsin-EDTA (Gibco, Grand Island, United States), washed in PBS and lysed in DNA extraction buffer [100 mM Tris–HCl pH 7.6, 100 mM NaCl, 10 mM EDTA, and 1% (w/v) N-lauroylsarcosine]. Lysates were digested with 50 μg/mL RNase A for 20 min at room temperature and then with 100 μg/mL proteinase K at 55°C for 12–16 h. DNA was extracted using three rounds of phenol/chloroform/isoamyl alcohol (25:24:1) solution (Sigma-Aldrich, Castle Hill, Australia) in MaXtract High Density tubes (Qiagen, Maryland, United States). DNA from the aqueous phase was then precipitated with 0.1 volume of 3 M sodium acetate pH 5.2 and 2.5 volumes of cold 100% ethanol. Finally, genomic DNA was washed with 70% ethanol, air dried and dissolved in 10 mM Tris–HCl pH 8.0, 1 mM EDTA. All subsequent assays (TCA, TRF, and qPCR) were carried out on the same sample of genomic DNA extracted from U-2 OS, HT1080, HT1080 hTR, HeLa, IIICF/c, and MRC-5 cells to minimize experimental variability.

Whole blood from healthy individuals and TBD patients was collected through venipuncture. For flow-fluorescence *in situ* hybridization (FISH), peripheral mononuclear blood cells (PBMCs) were isolated from whole blood using Histopaque-1077 (Sigma-Aldrich, Castle Hills, Australia) after the blood was homogenized with Hanks Balanced Salt Solution (Sigma-Aldrich, Castle Hills, Australia) to maintain pH and osmotic balance. For qPCR procedures, DNA from the whole blood of healthy individuals and TBD patients was extracted using QIAGEN QIAamp DNA Mini Kit (Qiagen, Maryland, United States) according to the manufacturer’s protocol.

### Telomere Length Combing Assay (TCA)

**FIGURE 1:**
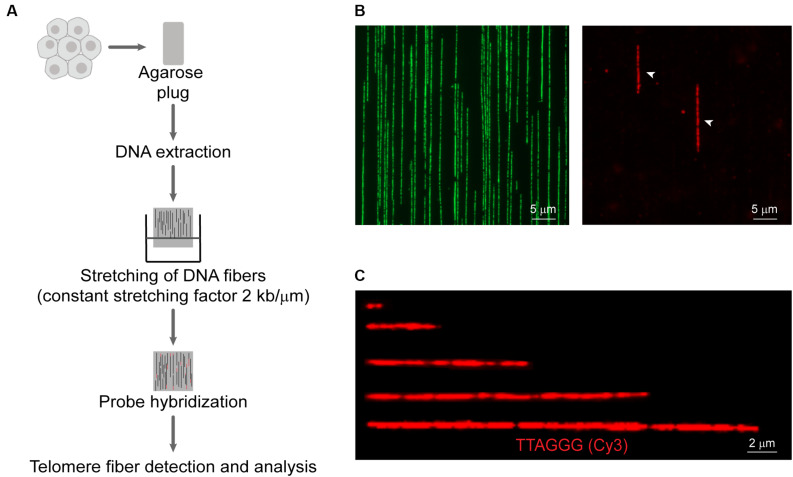
Overview of the Telomere length Combing Assay (TCA). **(A)** Cells are embedded in agarose plugs, prior to protein digestion. High molecular weight DNA is stretched onto vinylsilane coverslips and hybridized with a telomeric PNA probe. Telomeric fibers are then detected and measured. **(B)** Representative images of DNA counterstained by YOYO-1 (left panel) and telomere fibers (right panel). Telomere fibers are indicated by white arrows. Scale bars are 5 μm. **(C)** Examples of telomere fibers. Scale bars are 2 μm.

A detailed working protocol is provided in the Supplementary Material and an overview of the method is shown in Figure 1. Briefly, cells were isolated by trypsinization, embedded in agarose plugs (Sigma-Aldrich, Castle Hills, Australia), and subjected to proteinase K (0.5 M EDTA pH 8.0, 10% (v/v) sarcosyl/0.5 M EDTA, and 20 mg/mL proteinase K) digestion at 50°C for 12–16 h. Plugs were dissolved with agarase (Thermo Scientific, Norwood, Australia) for 12–16 h. The DNA solution was then transferred to a reservoir (Genomic Vision, Paris, France). Molecular combing was performed using the FiberComb^®^ Molecular Combing System (Genomic Vision, Paris, France) with a constant stretching factor of 2 kb/μm using vinylsilane coverslips (20 × 20 mm; Genomic Vision, Paris, France), according to the manufacturer’s instructions. Quality and integrity of stretched DNA fibers were checked using the YOYO-1^TM^ Iodide counterstain (Thermo Scientific, Norwood, Australia). Combed coverslips were incubated at 60°C for 4 h to minimize photobreaking, followed by serial ethanol dehydration (70–100%). Coverslips were hybridized with telomeric C- or G-rich PNA probe [TAMRA-OO-(CCCTAA)_3_ or TAMRA-OO-KK(TTAGGG)_3_] (Panagene, Daejeon, South Korea) in PNA hybridization buffer [70% (v/v) deionized formamide, 0.25% (v/v) NEN blocking reagent (PerkinElmer), 10 mM Tris–HCl (pH 7.5), 4 mM Na_2_HPO_4_, 0.5 mM citric acid, and 1.25 mM MgCl_2_] at room temperature for 12–16 h. Coverslips were washed and counterstained with YOYO-1^TM^ Iodide. Telomere fibers were detected on a Zeiss Axio Imager microscope and analyzed using ZEN v2.3 Pro software (Carl Zeiss, North Ryde, Australia).

### Terminal Restriction Fragment (TRF)

Terminal restriction fragments were obtained from genomic DNA by complete digestion with the restriction enzymes *Hin*fI and *Rsa*I. TRFs were separated by pulsed-field gel electrophoresis. Gels were dried, denatured and subjected to in-gel hybridization with a γ-[^32^P]-ATP-labeled (CCCTAA)_4_ oligonucleotide probe. Gels were washed and the telomeric signal visualized by PhosphorImage analysis (Conomos et al., 2012). TRFs were either visually compared or processed by MultiGauge image analysis software (Fuji Pharma) to quantitate mean telomere length.

### Quantitative PCR (qPCR)

Telomere qPCR was performed relative to the single copy gene 36B4 or HBG (Cawthon, 2002; Lee et al., 2014). Results were expressed relative to U-2 OS and presented as relative telomere content (arbitrary units). Experiments were carried out in a Rotor-Gene Q platform (Qiagen, Maryland, United States) and analyzed using Rotor-Gene 6000 series software (Qiagen, Maryland, United States).

### Quantitative-Fluorescence *in situ* Hybridization (Q-FISH)

Cells were treated with 10 μg/mL colcemid (Gibco, Grand Island, United States) for 3 h to arrest cells in metaphase, harvested by trypsinization, and resuspended in 75 mM KCl at 37°C for 20 min. Cells were fixed in methanol:acetic acid (3:1), and chromosome spreads were obtained according to standard cytogenetic methods. Microscope slides were subjected to serial ethanol dehydration (70–100%), followed by hybridization with 0.3 μg/mL Alexa-488-OO-(CCCTAA)_3_ telomeric PNA probe (Panagene, Daejeon, South Korea) at 80°C for 5 min, then at room temperature for 8 h. Slides were washed at 43°C for 5 min with each of the following solutions: 50% formamide in 2xSSC; 2xSSC; and 0.1% Tween-20/2xSSC. Slides were mounted using ProLong^TM^ Gold antifade with DAPI counterstaining (Invitrogen, Norwood, Australia). One hundred and fifty metaphases from each cell line were scored using MetaSystems software (MetaSystems, North Ryde, Australia). Results were shown as mean telomere intensity (arbitrary units × 1000).

### Flow-Fluorescence *in situ* Hybridization (Flow-FISH)

Equal numbers (3 × 10^6^) of control CCRF-CEM cells were mixed with experimental cells prior to denaturation and overnight hybridization at room temperature with 0.3 nM FITC-OO-(CCCTAA)_3_ telomeric PNA probe (Panagene, Daejeon, South Korea). Unstained duplicate tubes were run in parallel to determine autofluorescence. Cells were washed twice (70% formamide, 0.1% Tween-20), once (PBS, 0.1% Tween-20), followed by incubation at 4°C for 3 h with 500 μL of PBS containing 0.1% BSA, RNase A, and DNA binding dye LDS-751 (Santa Cruz Biotech, CA, United States). Data were acquired using FACS-Canto (BD Biosciences, NJ, United States) by acquisition of 1 × 10^4^ cells (with and without PNA probe incubation) to calculate mean fluorescence intensity (FI). Data were analyzed using FACSDiva^TM^ software (BD Biosciences, NJ, United States). Cells were gated to include the tetraploid CCRF-CEM cells. Relative telomere length (RTL) was calculated using the following formula: RTL = (mean FI of experimental cells with PNA probe) – (mean FI of experimental cells without PNA probe)/(mean FI of CCRF-CEM cells with PNA probe) – (mean FI of CEM cells without PNA probe). Results were shown as RTL.

### Semi-Automated Image Analysis for TCA

Two user-friendly semi-automated pipelines were developed using free, open-source platforms for quantification of TCA: CellProfiler v2.2.0 (Jones et al., 2008) and ImageJ v1.8.0 for Windows (NIH, Bethesda, MD, United States). Overall, both pipelines use a similar general approach for semi-automatic detection and size annotation of telomere fiber signals. However, some program-specific adaptations were made to improve the accuracy of telomere fiber identification.

#### CellProfiler Pipeline

Image manipulation (*tubeness*) was applied to smooth telomere fibers and reduce background. Telomere fibers were subjected to a maximum correlation threshold (MCT) algorithm and selected based on the following parameters: (1) ratio between major and minor axis > 2; (2) eccentricity ≥ 0.75; (3) width > 5 and < 15 pixels; (4) orientation of the major axis of the object is ± 15° of vertical relative to *X*-axis of the image. Telomere fibers that met the selection criteria were refined further by closing small gaps along the length of the fiber. Finally, telomere fiber identification was manually checked for accuracy and corrected, if necessary, using the manually edit objects function.

#### ImageJ Pipeline

Prior to telomere identification a separate pipeline was used to create a binary mask of the image for background detection and refinement. This involved applying a series of open shape selector indicators as disk, horizontal lines and the 45° and 145° lines, using the MorphoLibJ plugin (v1.4.1). Next, a *tubeness* processing filter was used to smooth telomere fibers and reduce background. Telomere fibers were then subjected to a user-defined threshold (chosen with a slider bar) and a binary mask was created. Finally, the background mask generated in the first pipeline was subtracted from the newly generated telomere mask image and telomere fibers were further selected by the following parameters: (1) size > 0.250 μm^2^; (2) circularity from 0 to 0.5. Telomere fibers that met the selection criteria were refined further by closing small gaps along the length of the fiber (MorphoLibJ plugin, v1.4.1).

### Automated Image Analysis for TCA Using the Molecular Combing Platform

The FiberVision^®^ scanner (Genomic Vision, Paris, France) was used to acquire multi-color images of the whole surface of combed coverslips. FiberStudio^®^ software (Genomic Vision, Paris, France) was used to detect and measure telomere fiber signals using a custom-designed algorithm. Samples were prepared as described in the Telomere length Combing Assay (TCA) section, with minor modifications. First, coverslips were hybridized with telomeric C-rich PNA probe (Alexa647-OO-(CCCTAA)_3_; 0.3 μg/mL; Panagene, Daejeon, South Korea). Second, for imaging and analysis purposes, coverslips were mounted in barcoded cartridges (Genomic Vision, Paris, France), and loaded onto the FiberVision^®^ scanner. Third, images were automatically acquired at 40×, imported into FiberStudio^®^ software, and automatically scored. FiberStudio^®^ software allows the scoring to be reviewed, and generates a report containing the telomere length distribution for all regions of interest (ROI).

### Statistics

Graphs and statistical analysis were generated using GraphPad Prism v5.04. The two-sided Student’s *t*-test was performed on normally distributed data, while the two-sided Mann-Whitney test was performed on data assumed to be non-normally distributed. Linear regression was used to test the correlation between datasets. Further details of statistical analyses are provided in the figures and figure legends.

## Results

### Molecular Combing Can Be Used to Measure Telomere Length

Molecular combing, or DNA fiber analysis, is a powerful technique used to visualize genomic loci and repetitive sequences in the genome (Parra and Windle, 1993). DNA fiber analysis involves extracting DNA from cells such that the chromatin is deproteinized leaving naked DNA, which is then stretched onto a glass microscope slide. This can be combined with FISH for the detection and measurement of specific genomic sequences (Ersfeld, 1994). More recently, DNA fiber analysis has been used to study replication dynamics by tracking the sequential incorporation of halogenated nucleotides into replicating DNA (Norio and Schildkraut, 2001). This technique has been further adapted to analyze the progression of replication forks through telomeric DNA (Sfeir et al., 2009), as well as for the measurement of telomere extension events (Sobinoff et al., 2017; Lu et al., 2019).

An underexplored application of molecular combing is the direct measurement of telomere length (Wang et al., 2013). More recently, a CRISPR-Cas9 nickase system has been used to label telomere tracts, followed by nanochannel array analysis to measure discrete telomere lengths (McCaffrey et al., 2017). We have developed the TCA to measure the distribution of individual telomere lengths in a cell population. A schematic representation of TCA is presented (Figure 1A), and a detailed protocol is provided (Supplementary Material). To preserve the integrity of the telomeric DNA and to minimize shearing, cells were embedded in agarose plugs, prior to protein digestion to release the DNA from the chromatin scaffold. The DNA was then solubilized. Vinylsilane treated glass coverslips were submerged in the solution and withdrawn mechanically, allowing the DNA molecules to be stretched at a constant stretching factor of 2 kb/μm. The utilization of a constant stretching factor that is both irrespective of sequence content and DNA fiber length, and uniform across the glass surface (Schurra and Bensimon, 2009), obviates the requirement to normalize the fiber length internally, providing that the stretching dynamics are regularly calibrated. DNA fibers were then hybridized to a telomere-specific PNA probe and subjected to DNA counterstaining. The YOYO-1 counterstain was used to provide additional information pertaining to the terminal location of the telomere, thus minimizing the inclusion of interstitial repeat arrays or extra-chromosomal telomeric repeats (ECTRs) in the telomere length measurements. Finally, telomere fibers were detected by fluorescence microscopy (Figure 1B), and the length of the telomere repeat tracts measured using the associated microscope software, or by automated image analysis (Figure 1C). TCA was able to accurately measure telomere lengths in human cells or extracted DNA within a range of <1 kb and >80 kb. TCA is a simple and robust technique to measure the telomere lengths of individual DNA molecules, providing the distribution of telomere lengths in a cell population.

### Comparison of TCA With Established Telomere Length Measurement Techniques

**FIGURE 2:**
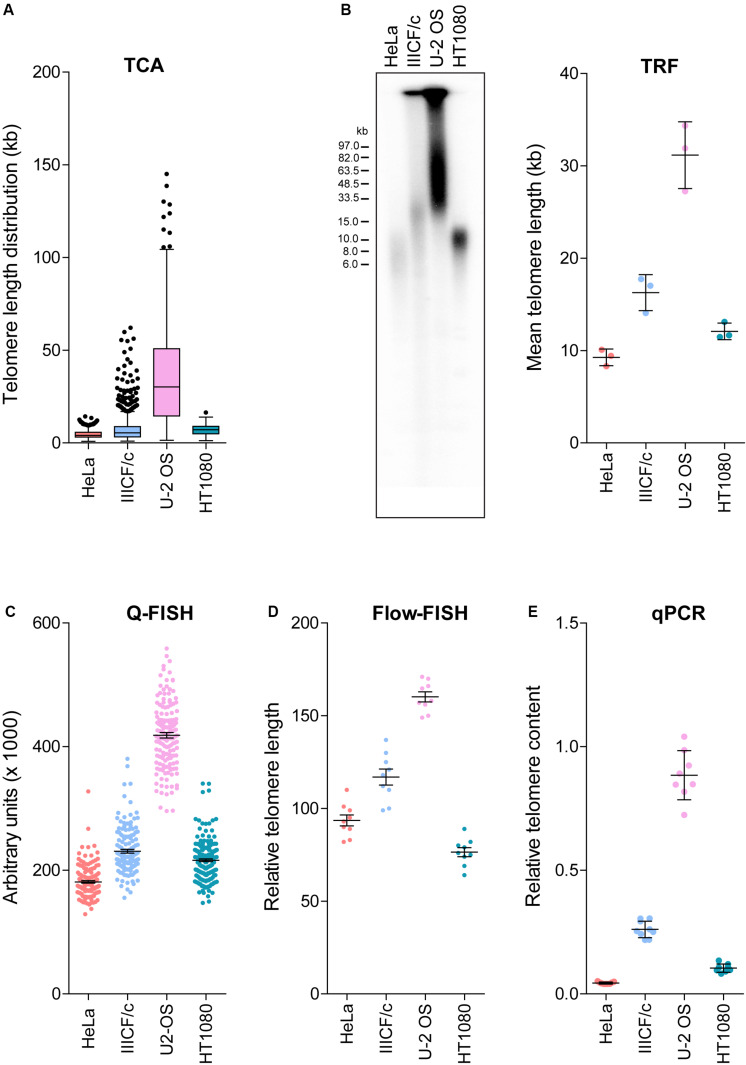
Comparison of telomere length measurement methods. **(A)** Distribution of individual telomere lengths measured by TCA in HeLa (telomerase-positive), IIICF/c (ALT), U-2 OS (ALT) and HT1080 (telomerase-positive) cell lines. Tukey boxplots of median from >450 telomeric fibers from *n* = 3 technical replicates. **(B)** TRF analysis of telomere length (left panel) and mean telomere length quantitated using MultiGauge image analysis software (right panel). Error bars are mean ± SEM from *n* = 3 independent experiments. **(C)** Telomere length measurements by Q-FISH. Results are presented as arbitrary units (×1000). Error bars are mean ± SEM from ≥3,000 telomere signals. **(D)** Telomere length measurements by flow-FISH. Results are presented as relative telomere length (RTL). Error bars are mean ± SEM from *n* = 3 independent experiments with 3 technical replicates each. **(E)** Telomere content analyzed by qPCR relative to the single copy gene 36B4 and normalized to the U-2 OS reference sample. Error bars are mean ± SEM from *n* = 3 independent experiments with 3 technical replicates each.

Established telomere length measurement techniques have various strengths and limitations (Aubert et al., 2012; Montpetit et al., 2014; Lai et al., 2018), with most techniques providing a relative or average measurement of length or telomere content. TCA overcomes this limitation by measuring individual telomeres. We measured the distribution of telomere lengths by TCA in four different cell lines (Figure 2A). These cell lines were selected based on their well-established differences in telomere length and TMM, with HeLa, and HT1080 having short telomeres and utilizing telomerase as the TMM, and IIICF/c and U-2 OS having longer and heterogeneous telomere lengths and utilizing the ALT pathway of telomere maintenance (Supplementary Table S1; Lee et al., 2014). TCA demonstrated different telomere length distribution profiles in the four different cell lines. Mean telomere length measurements were 4.55 kb for HeLa (min: 0.96 kb; max: 14.29 kb), 7.60 kb for IIICF/c (min: 0.87 kb; max: 62.09 kb), 36.11 kb for U-2 OS (min: 1.41 kb; max: 145.10 kb), and 6.95 kb for HT1080 (min: 1.82 kb; max: 16.46 kb; Figure 2A). Telomere length analysis by TCA further identified the presence of very long (>80 kb) as well as very short (<1 kb) telomere lengths in U-2 OS and IIICF/c cells, consistent with these cell lines utilizing the ALT mechanism of telomere maintenance (Figure 2A). This contrasted with the short homogeneous telomere lengths observed in the two telomerase-positive cell lines (HeLa and HT1080).

We then compared TCA to the most commonly utilized telomere length measurement techniques in the same four cell lines (Figures 2A–E). TRF analysis is the gold standard telomere length measurement technique employed by molecular biology labs (Kimura et al., 2010; Mender and Shay, 2015). TRF analysis was used to visualize the intensity and size distribution of TRFs (Figure 2B), and the mean telomere length from the contributing cell population was estimated from the size of the heterogeneous telomere smear (Figure 2B). Quantitative-FISH (Q-FISH) can be applied to interphase or metaphase cells to provide high resolution measurements of individual telomere lengths (Lansdorp et al., 1996; Lai et al., 2018), while flow-FISH has been extensively validated for clinical diagnostic purposes and employs flow cytometry to detect telomere-bound fluorescently labeled probes in individual viable cells (Rufer et al., 1998; Figures 2C,D). Quantitative PCR (qPCR) has been broadly adopted for clinical and epidemiological studies, and benefits from its technical simplicity and the requirement for small amounts of DNA, but produces variable results (Cawthon, 2002; Lai et al., 2018). qPCR of amplified telomeric products was compared to a reference single copy gene, to provide a measure of relative telomere content in the four cell lines (Figure 2E).

Overall, we found that telomere lengths were comparable across all five techniques (Figures 2A–E), with the U-2 OS cell line consistently displaying the longest and most heterogeneous telomere lengths. The HeLa and HT1080 cell lines consistently displayed short telomere lengths. Some variability regarding the cell line with the shortest telomeres was observed, with flow-FISH indicating that HT1080s had the shortest telomere lengths, in contrast to all other methods, which identified HeLa as having the shortest telomeres. Variability in the RTL of the four cell lines was also observed between methods. TCA and Q-FISH were the only techniques employed here that measure individual telomere lengths. The distribution of telomere lengths measured by TCA and Q-FISH were further compared using telomere length frequency histograms, with TCA demonstrating increased resolution of very long telomeres in the two ALT cell lines compared to Q-FISH (Supplementary Figure S1). By directly comparing telomere lengths measured by TCA to measurements made using the other methods, the most tightly correlated techniques were TCA and qPCR (*R*^2^ = 0.93), followed by TCA and Q-FISH (*R*^2^ = 0.91), TCA and TRF (*R*^2^ = 0.80), and TCA and flow-FISH (*R*^2^ = 0.78).

We then aimed to determine the accuracy and reproducibility of TCA, and its versatility to different sample preparations. TCA is typically performed on freshly harvested cells embedded in agarose plugs and in-gel protein digestion to minimize DNA shearing, prior to stretching of DNA fibers. We compared embedded cells versus phenol-chloroform extracted DNA from U-2 OS and HeLa cell lines and from the MRC-5 mortal cell strain (Supplementary Figure S2). Telomere length measurements were comparable for HeLa and MRC-5 cells, which have short telomere lengths, whilst significant variability in telomere length was observed between the extracted DNA samples compared to the cell preparation samples for U-2 OS cells (Supplementary Figure S2). Specifically, telomere length distributions revealed an under-representation of the longest telomeres in U-2 OS cells, indicative of some level of DNA shearing. This suggests that, whilst TCA is a reproducible and versatile technique, the requirement for embedded cells is more critical when analyzing cells with very long telomeres, in contrast to shorter telomeres, which are less sensitive to DNA processing. This is unlikely to be a concern when analyzing human samples, as only ALT cells with extremely long telomeres were significantly impacted by the skewed representation. Overall, these data support the utility of TCA as an accurate and robust technique to measure the distribution of individual telomere lengths in a cell population.

### TCA Can Be Used to Measure Dynamic Changes in Telomere Length

**FIGURE 3:**
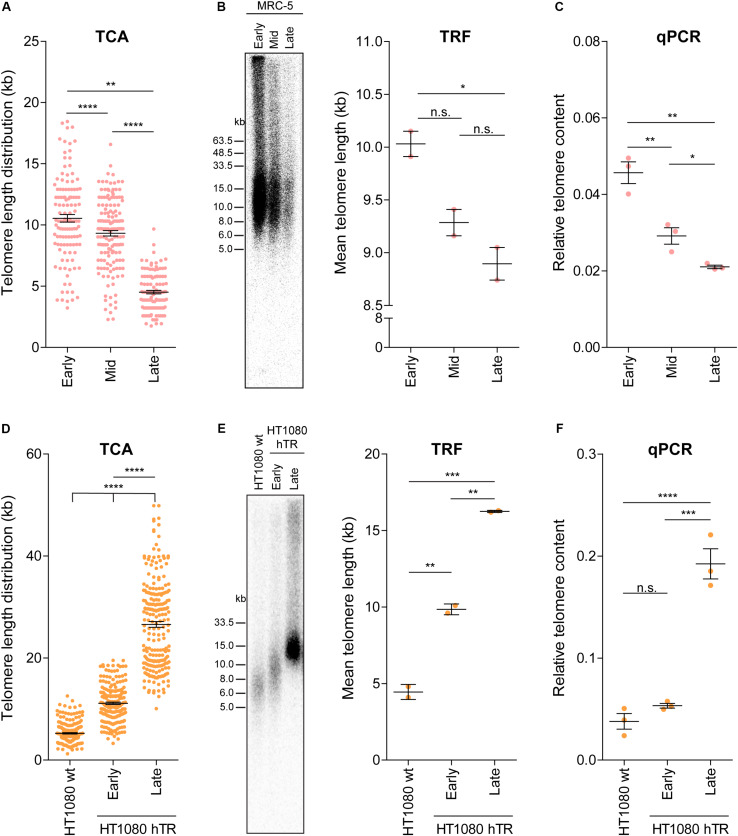
TCA can accurately measure telomere attrition and telomerase-mediated telomere extension. **(A)** Telomere length measurements in MRC-5 fibroblasts at early (20), mid (40), and late (60) population doublings (PDs). Scatterplots showing the distribution of ≥150 individual telomere fiber lengths measured by TCA. Error bars represent the mean ± SEM from *n* = 3 technical replicates, **p < 0.01 and ****p < 0.0001. Student’s *t*-test. **(B)** TRF analysis (left panel) and quantitation using MultiGauge image analysis software (right panel). Error bars represent the mean ± SEM from *n* = 2 technical replicates, *p < 0.05 and n.s. *p* > 0.05. Student’s *t*-test. **(C)** Telomere content analyzed by qPCR relative to the single copy gene 36B4 and normalized to the U-2 OS reference sample. Error bars represent the mean ± SEM from *n* = 3 technical replicates, *p < 0.05 and **p < 0.01. Student’s *t*-test. **(D)** Telomere length measurements in HT1080 wild type (wt) and HT1080 hTR at early (10) and late (90) PDs. Scatterplots showing the distribution of ≥150 individual telomeric fiber lengths measured by TCA. Error bars represent the mean ± SEM from *n* = 3 technical replicates, ****p < 0.0001. Student’s *t*-test. **(E)** TRF analysis (left panel) and quantitation using MultiGauge image analysis software (right panel). Error bars represent the mean ± SEM from *n* = 2 technical replicates, **p < 0.01 and ***p < 0.001. Student’s *t*-test. **(F)** Telomere content analyzed by qPCR relative to the single copy gene 36B4 and normalized to the U-2 OS reference sample. Error bars represent the mean ± SEM from *n* = 3 technical replicates, ***p < 0.001, ****p < 0.0001, and n.s. *p* > 0.05. Student’s *t*-test.

Telomere attrition and TMMs contribute to telomere length regulation, and telomere length ultimately dictates the proliferative capacity of a cell. To address whether TCA provides sufficient sensitivity to detect dynamic changes in telomere length, we used three separate cell systems. First, we used primary human fibroblasts that undergo telomere attrition at a rate of 50–150 bp per cell division (Harley et al., 1990), until telomere lengths become critically short and the cell reaches senescence. Cellular senescence is characterized by an absence of mitotic cell division and permanent disengagement from the cell cycle (Reddel, 2000). TCA was used to measure telomere lengths in the MRC-5 mortal human lung fibroblast cell strain at early, mid and late population doublings (PDs; Figure 3A). Telomere shortening was observed with increasing PDs. Specifically, early and mid PDs demonstrated mean telomere length measurements of 10.55 kb and 9.32 kb, respectively. The late timepoint (PD 60) corresponded with the approximate onset of cellular senescence (Kaul et al., 2012), and had a mean telomere length of 4.52 kb. This demonstrates the sensitivity of TCA to accurately determine telomere length changes of ≥1 kb. Absolute telomere length measurements were used to calculate a rate of attrition of approximately 70 bp/cell division, consistent with normal rates of telomere attrition in cells lacking a TMM. Importantly, TCA is not dependent on mitotic cells for the measurement of individual telomere lengths, allowing senescent cells to be measured by TCA.

Telomere length measurements by TCA were then compared to telomere lengths measured by TRF analysis and by qPCR in MRC-5 cells at early, mid and late PDs (Figures 3B,C). All three techniques demonstrated telomere shortening with increasing PD (Figures 3A–C); however, the difference in telomere length by TRF was only significant between early and late PD, indicative of the lower sensitivity of TRF analysis to detect dynamic changes in telomere length (Figure 3B). Interestingly, absolute telomere length measurements at early and mid-timepoints were similar for TCA and TRF analysis. These two techniques provide a measure of telomere length in kb, although TRFs include variable amounts of subtelomeric regions, dictated by the position of the terminal restriction enzyme site. At the later timepoints telomere lengths appeared shorter when measured by TCA compared to TRF analysis, supporting TCA being the more sensitive technique (Figures 3A,B).

Second, we used the previously described HT1080 hTR super-telomerase cell line to measure telomerase-mediated telomere lengthening (Pickett et al., 2009). HT1080 hTR cells that exogenously express hTR display elevated levels of telomerase activity and progressive telomere lengthening over 150 PDs, until telomere lengths plateau (Pickett et al., 2009). TCA identified progressive telomere lengthening in the HT1080 hTR cell line at early (mean telomere length: 11.12 kb) and late (26.59 kb) PDs compared to the parental HT1080 wild-type (5.25 kb) cell line, with the late timepoint corresponding to approximately 90 PDs post stable overexpression of hTR (Figure 3D). Telomere lengthening was similarly observed by TRF analysis and by qPCR (Figures 3E,F). TCA was able to provide a more detailed distribution profile of telomere lengths than the telomere length measurements obtained by TRF and qPCR analysis. This was most striking at the later timepoints when telomere lengths were longer and more heterogeneous (Figure 3D).

Third, we used TCA to measure the telomere length changes that occur in ALT cells in response to manipulation of SLX4 and BLM levels (Sobinoff et al., 2017). Specifically, we used SLX4 overexpression to promote resolution of telomere recombination intermediates, thereby causing telomere shortening, and BLM overexpression to promote ALT-mediated telomere extension (Sobinoff et al., 2017). Telomere length distribution profiles obtained by TCA demonstrated telomere shortening in response to SLX4 overexpression, and telomere lengthening in response to BLM overexpression in IIICF/c (mean telomere lengths: empty vector: 14.24 kb; BLM + : 20.77; and SLX4 + : 10.92), and U-2 OS (empty vector: 22.73 kb; BLM + : 25.91; and SLX4 + : 12.30) ALT cell lines, but not in HT1080 hTR telomerase-positive cells (empty vector: 10.05 kb; BLM + : 10.92; and SLX4 + : 10.79; Supplementary Figure S3), consistent with previous reports (Sobinoff et al., 2017). Notably, TCA was able to provide more precise measurements of the distribution of individual telomere lengths, identifying a striking decrease in long telomeres in the SLX4 overexpressing cells, and a noticeable increase in very long telomeres in the BLM overexpressing cells (Supplementary Figure S3). Overall, these data demonstrate that TCA can accurately measure dynamic changes in telomere length, including normal telomere attrition in primary human cells, and both telomerase- and ALT-mediated telomere extension in cancer cells.

### TCA Can Be Used to Measure Telomere Length in the Human Population

**FIGURE 4:**
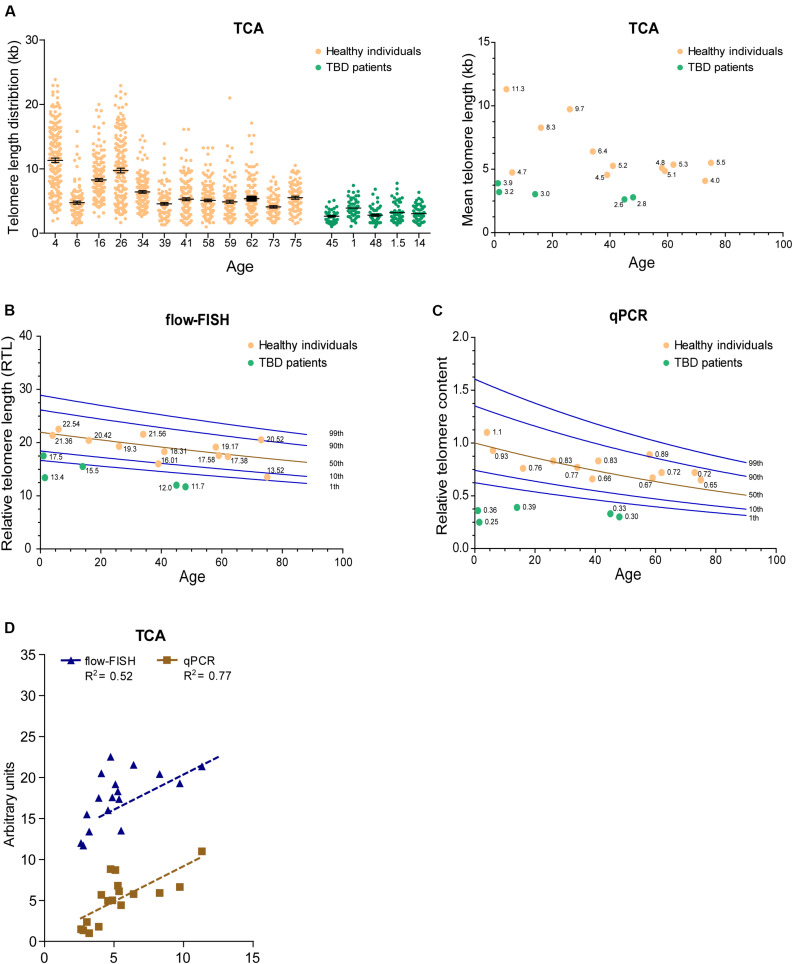
TCA can be used to measure telomere length in the human population. **(A)** Telomere length measurements in twelve healthy individuals spanning ages 4 to 75 years and five TBD patients. Scatterplots showing the distribution of ≥150 individual telomeric fiber lengths measured by TCA. Error bars are mean ± SEM from *n* = 2 technical replicates (left panel). Mean telomere length determined by TCA plotted against age for healthy individuals and TBD patients. Annotated numbers indicate mean telomere length (kb; right panel). **(B)** RTL measured by flow-FISH plotted against age for healthy individuals and TBD patients. Lines indicate the 1st, 10th, 50th, 90th, and 99th telomere length percentiles from a cohort of 240 normal controls. **(C)** Telomere content analyzed by qPCR. Ratio between telomere content and single copy gene HBG plotted against age for healthy individuals and TBD patients. Lines indicate the 1st, 10th, 50th, 90th, and 99th telomere length percentiles from a cohort of 240 normal controls. **(D)** Linear regression of TCA against flow-FISH (*R*^2^ = 0.52) and qPCR (*R*^2^ = 0.77) for healthy individuals and TBD patients. Spearman correlation test.

Telomere length decreases with advancing age and is considered a biomarker of chronological aging. Nevertheless, inter-individual telomere lengths at any given age are highly variable in the human population (Harley et al., 1990; Aubert and Lansdorp, 2008). To determine whether TCA can detect age-associated decline in telomere length, we accessed DNA extracted from peripheral blood mononuclear cells (PBMCs) from twelve healthy individuals spanning ages 4 to 75 years of age, collected as part of a cohort of 240 healthy individuals used to obtain normal telomere length percentiles for different ages through the Department of Hematology Telomere Length Testing Facility, Sydney Children’s Hospitals Network, Australia. TCA provided a distribution of telomere lengths for each individual, demonstrating an overall decrease in telomere length with chronological age (Figure 4A).

Telomere biology disorders are premature aging diseases caused by abnormally short telomeres, with lengths typically falling below the bottom percentile of the normal healthy population. The clinical characteristics of TBDs are diverse, but patients frequently develop bone marrow failure and idiopathic pulmonary fibrosis (Mangaonkar and Patnaik, 2018). Telomere length analysis is used to identify individuals suspected of having TBDs, but can also provide prognostic information regarding disease onset and clinical manifestations, as well as directing treatment regimens (Alder et al., 2018). We used TCA to measure the telomere lengths of five individuals (ages 1, 1.5, 14, 45, and 48 years old) that had previously been diagnosed with TBDs, using DNA extracted from PBMCs. TCA analysis showed that these individuals all had strikingly short telomere length distribution profiles, and that their mean telomere lengths were substantially shorter than those of healthy individuals (Figure 4A).

We then compared telomere length measurements by TCA to telomere lengths measured by flow-FISH and qPCR for the healthy individuals and the TBD patients. Normal telomere length percentiles for the cohort of 240 healthy individuals were included for the flow-FISH and qPCR datasets (Figures 4B,C). Telomere lengths for the healthy individuals were somewhat variable across the three different techniques, but an overall decline in telomere length with chronological age was always observed (Figures 4A–C). TCA correlated well with qPCR (*R*^2^ = 0.77), but not as well with flow-FISH (*R*^2^ = 0.52), for this specific dataset (Figure 4D). This is consistent with previous reports that indicate variable correlations between different telomere length measurement methods (Khincha et al., 2017). All three techniques identified the TBD patients as having abnormally short telomeres (Figures 4A–C), falling below the tenth percentile of the healthy cohort. These data demonstrate that TCA can be used to measure telomere lengths in PBMCs from healthy individuals. Importantly, TCA can reliably identify patients with critically short telomere lengths, supporting a potential practical application for TCA in the diagnosis and treatment of TBDs.

### Software Automation for TCA Quantification

Detection and measurement of telomere fibers was performed manually using image manipulation tools. Each technical replicate generated an average of 300 individual telomere fibers to be scored. In order to adapt TCA for high-throughput applications, we developed pipelines for the semi-automated detection and size annotation of telomere lengths using the open-source image processing platforms CellProfiler and ImageJ, and we evaluated fully automated telomere length measurement using FiberStudio^®^ automated analysis and reporting software, in collaboration with Genomic Vision (France).

The detection and analysis pipelines for CellProfiler and ImageJ are presented (Supplementary Figures S4A,B). Tubeness processing allows both CellProfiler and ImageJ algorithms to close small gaps along the identified telomeric fiber. Both CellProfiler and ImageJ provide an optional step for the user to check and modify identified fibers. This was employed here to validate the pipelines. Cell Profiler and ImageJ pipelines require manual imaging of the telomere fibers through microscope tiling image manipulation tools and are, therefore, semi-automated pipelines. In contrast, the FiberVision^®^ scanner automatically images the slide, and custom-designed FiberStudio^®^ software detects, quantifies, and annotates the telomere fibers, providing a fully automated platform.

**FIGURE 5:**
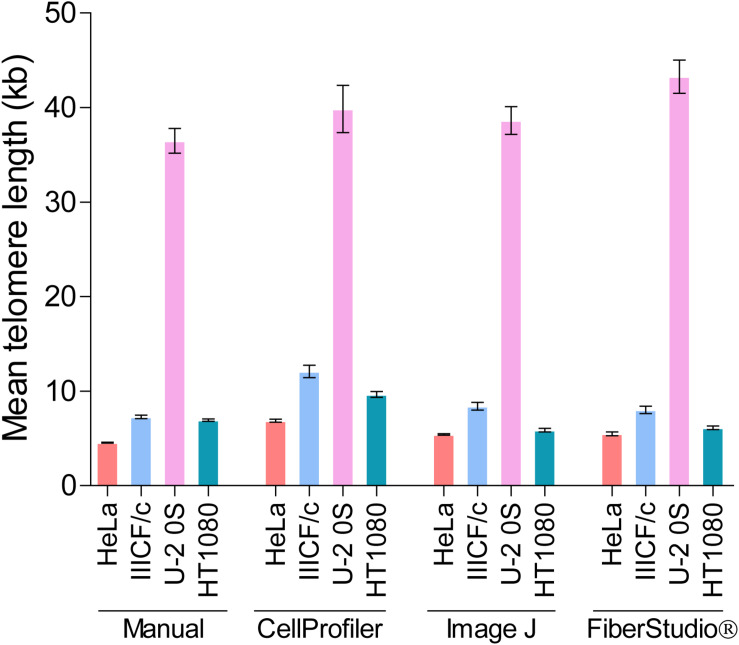
Mean telomere length (kb) measured by TCA in HeLa, IIICF/c, U-2 OS, and HT1080 cell lines using manual measurement tools compared to the CellProfiler and ImageJ semi-automated image analysis pipelines and to FiberStudio^®^ automated software.

While differences in image manipulation and telomere signal detection exist between the two semi-automated programs, both CellProfiler and ImageJ performed similarly, and produced comparable measurements of mean telomere fiber length to the measurements obtained from manual analysis in the HeLa, IIICF/c, U-2 OS, and HT1080 cell lines (Figure 5). FiberStudio^®^ also demonstrated accuracy in measuring telomere lengths from HeLa, IIICF/c, U-2 OS, and HT1080 cell lines when compared to manual scoring (Figure 5).

To demonstrate the accuracy of TCA, we measured the same fifty fibers from HeLa cells three separate times. Measurements were made manually, and each replicate was scored independently. Telomere length measurements demonstrated a very high level of accuracy between replicates (Supplementary Figure S5A).

Finally, to determine the potential utility of automated TCA analysis, we directly compared the lengths of fifty telomere fibers from HeLa cells measured manually to the lengths of the same fifty telomere fibers measured using CellProfiler, ImageJ, and FiberStudio^®^. The individual telomere length measurements obtained by manual assessment correlated similarly with those obtained using CellProfiler (*R*^2^ = 0.80) and ImageJ (*R*^2^ = 0.81), and correlated slightly better with measurements obtained using FiberStudio^®^ (*R*^2^ = 0.84; Supplementary Figure S5B). These results demonstrate that TCA is suitable for both semi-automated and fully automated image analysis. The successful application of CellProfiler, ImageJ, and FiberStudio^®^ to the detection and accurate measurement of telomere fibers, with high correlation to manual measurements, demonstrates the utility of these programs in providing an unbiased approach for the detection and measurement of individual telomere fibers, with potential high-throughput capabilities.

## Discussion

We have developed TCA as a simple and accessible method to accurately measure the distribution of individual telomere lengths in a cell population. TCA involves stretching DNA fibers onto coated glass coverslips using a constant stretching factor. This achieves unbiased and uniform stretching of millions of DNA molecules, which can then be probed with telomere-specific PNA probes for the detection and measurement of individual telomere lengths. The major strengths of TCA are its simplicity, adaptability to image automation for high-throughput purposes, lack of requirement for cycling cells, and ability to accurately detect all telomere lengths in a cell population, including very short (<1 kb), and very long (>80 kb) telomeres. The main limitations of TCA are that it is relatively time-consuming and expensive (approximately twice the cost of flow-FISH), and relies on specialized molecular combing equipment and consumables.

By directly comparing TCA to other widely utilized telomere length measurement techniques (TRF, Q-FISH, flow-FISH, and qPCR), we demonstrated that TCA is sensitive and accurate for telomere detection and length measurement. TCA provides important and relevant information regarding the distribution of individual telomere lengths in a cell population. This is not achievable using current methods, which include TRF for molecular research, flow-FISH for clinical telomere length measurement applications and the diagnosis of TBDs, and qPCR for large-scale clinical and epidemiological applications. Other techniques, including Single TElomere Length Analysis (STELA), universal STELA (U-STELA; Bendix et al., 2010), and Telomere Shortest Length Assay (TeSLA; Lai et al., 2017), are able to detect individual telomere lengths, but include a PCR step that precludes the amplification and detection of very long telomeres, and are somewhat limited by their technical complexity (Lai et al., 2017, 2018).

TCA is versatile and can be applied reliably to both freshly harvested cells and extracted DNA, although the use of agarose-embedded cells is preferable for the measurement of very long telomeres to prevent shearing through mechanical manipulation. The requirement for isolated DNA as the starting material means that telomere length measurements by TCA are not dependent on cycling cells, in contrast to flow-FISH and Q-FISH methods. This is particularly important for the analysis of cells with a low mitotic index or cells approaching or at senescence (Kaul et al., 2012). In addition, the removal of nucleosomes by protease digestion leaves the DNA naked and amenable for stretching. This means that telomere length measurements are not affected by aberrant chromatin compaction that has the propensity to influence the binding of telomere FISH probes (Bandaria et al., 2016; Timashev et al., 2017; Vancevska et al., 2017).

TCA provides absolute measurements of individual telomere lengths, rather than an estimate of RTL or content. The telomere length distribution profile is representative of the range of telomere lengths in the cell population, and absolute measurements provide precise information regarding telomere length. In this study, we were able to measure telomere lengths as short as 0.87 kb and as long as 145.1 kb. The proportion of very short or very long telomeres in a cell population is biologically relevant (Aubert and Lansdorp, 2008; Baird, 2008a,b; Muntoni et al., 2009; Stanley and Armanios, 2015), but is typically not considered in clinical, epidemiological and research-based investigations. TCA provides the means to interrogate telomere length associations in far more detail than currently achievable.

TCA is sufficiently sensitive to detect dynamic changes in telomere length. Specifically, we measured telomere attrition in primary human fibroblasts, telomere lengthening in response to increased telomere maintenance by telomerase or ALT, and telomere shortening following the inhibition of telomere maintenance by ALT. TCA provided additional biological insight into these processes by uncovering the changes that occurred at the telomere length extremes, as well as identifying the overall shift in individual telomere length distribution profiles. The sensitivity of TCA to detect these intricacies will provide a more complete understanding of how telomere length drives replicative senescence and tumorigenic escape, as well as how inhibition of TMM pathways impacts telomere length and cell proliferation.

Telomere length measurements are increasingly being used as an indicator of lifetime health, to assess risk of many age-associated diseases, and to diagnose TBDs (Epel et al., 2004; Sampson and Hughes, 2006; Willeit et al., 2010; Haycock et al., 2014; Bertuch, 2016; Opresko and Shay, 2016; Khincha et al., 2017; Maciejewski and de Lange, 2017; Alder et al., 2018; Mangaonkar and Patnaik, 2018). We demonstrated that TCA can be used to measure age-associated telomere length in PBMCs isolated from healthy individuals spanning over 70 years and can accurately detect short telomeres in patients with TBDs. Interestingly, the telomere length distribution profiles obtained from patients with TBDs were very distinctive, showing very short and homogeneous telomere lengths. This supports the utility of TCA as an improved telomere length measurement technique for clinical testing and diagnosis.

We also demonstrated that TCA is readily amenable to automated image analysis. Specifically, we implemented two open-source software pipelines using CellProfiler and ImageJ, to semi-automatically measure the manually imaged TCA samples from HeLa, IIICF/c, U-2 OS, and HT1080 cell lines. Both pipelines were comparable to manual scoring. We then compared fully automated TCA using the FiberVision^®^ automated scanner and accompanying custom-designed FiberStudio^®^ software, and demonstrated an even higher correlation to manual scoring. Importantly, automation precludes bias and subjectivity, and enables rapid and accurate analysis of extensive datasets.

In summary, TCA is a simple and precise method for the measurement of telomere length. TCA has the potential to supersede currently available methods by fulfilling several advantages. First, TCA measurements provide additional relevant biological insight by measuring the distribution of individual telomere lengths, including both telomere length extremes. Second, TCA is versatile in terms of the sample preparation and starting material required. Third, TCA is adaptable to automated image analysis, which precludes bias and will facilitate high-throughput applications. TCA may be further improved by the inclusion of subtelomeric probes to enable the measurement of chromosome-specific telomeres, and the combined use of variant telomere repeat probes as an indicator of telomere directionality and integrity. Automated TCA represents an exciting and promising telomere length measurement technique for clinical and research applications.

## Data Availability Statement

All datasets generated for this study are included in the article/Supplementary Material.

## Ethics Statement

The studies involving human participants were reviewed and approved by Human Research Ethics Committee of the Sydney Children’s Hospitals Network. Written informed consent to participate in this study was provided by the participants’ legal guardian/next of kin.

## Author Contributions

HP, JA, and VK conceived the idea and designed the experiments. VK, JA, CN, AS, ML, TK, and RV performed the experiments and analyzed the data. HP and VK wrote the manuscript. All authors contributed to the manuscript and approved the submitted version.

## Conflict of Interest

The authors declare that the research was conducted in the absence of any commercial or financial relationships that could be construed as a potential conflict of interest.
