# Supplementary figures and images for: Telomere Length Measurement by Molecular Combing
Source: Front Cell Dev Biol. 2020 Jun 16;8:493. doi: 10.3389/fcell.2020.00493 (PMC7308456; doi:10.3389/fcell.2020.00493)

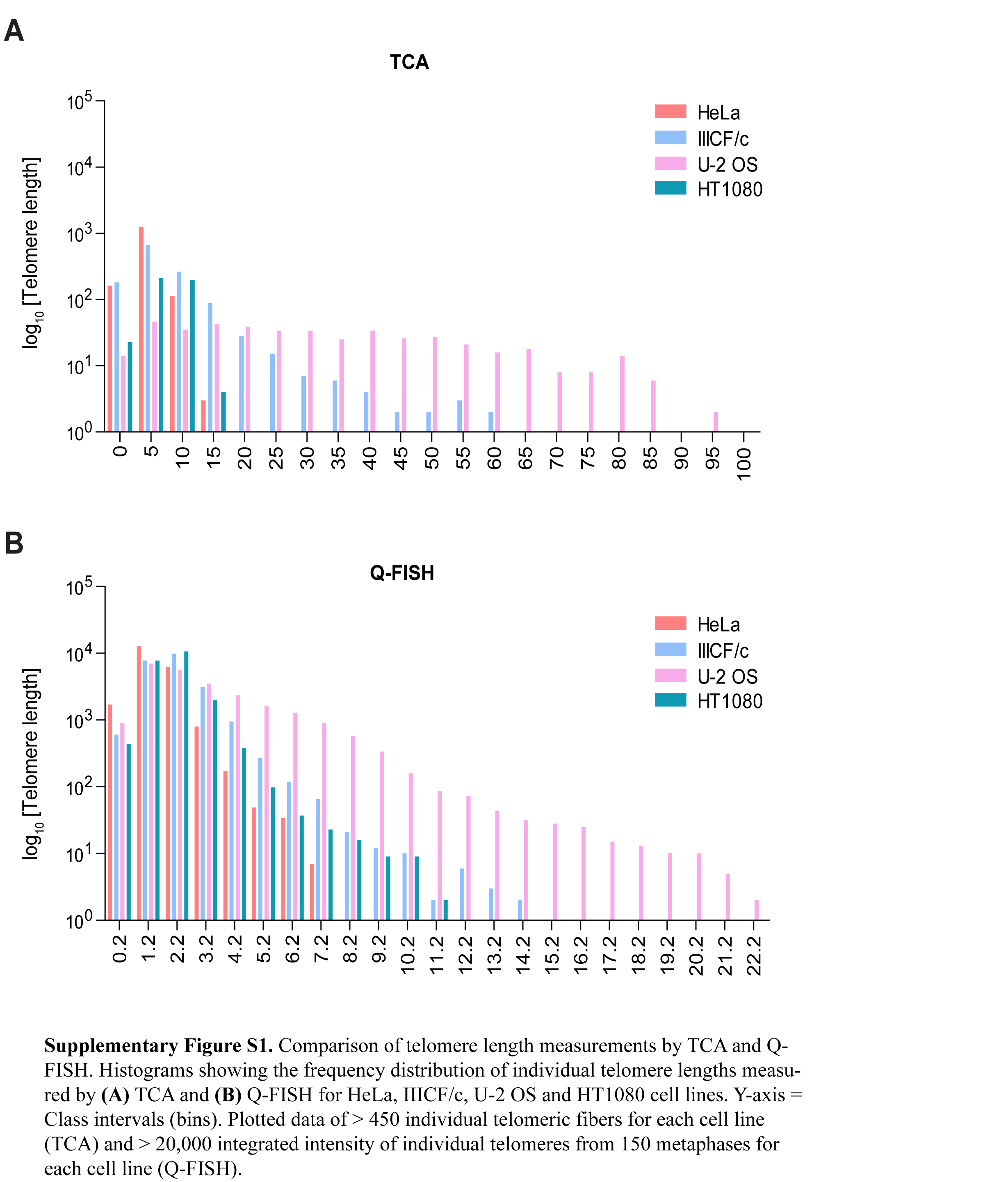

Supplement: Supplementary file 1 [file Image_1.tif]

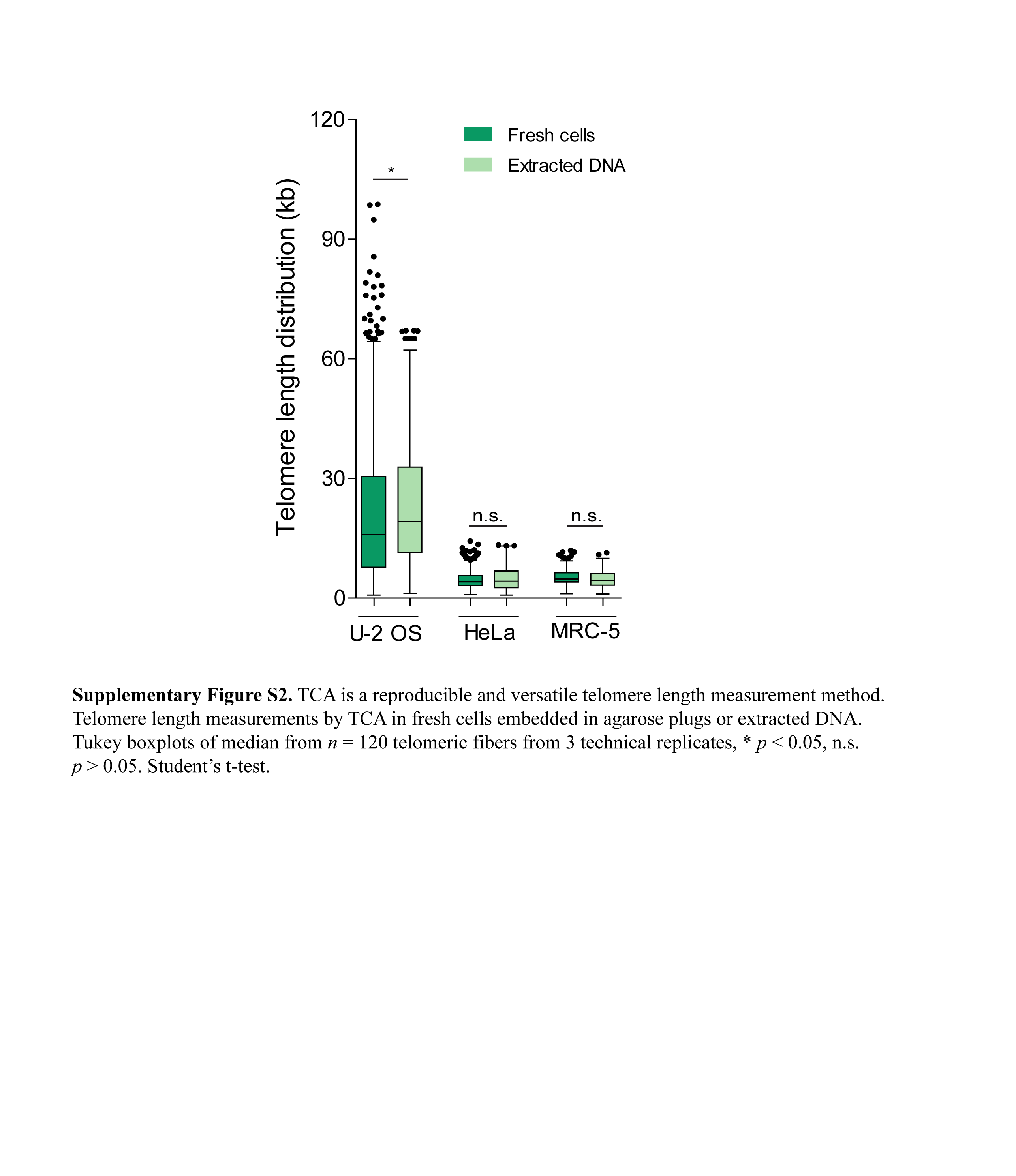

Supplement: Supplementary file 2 [file Image_2.tif]

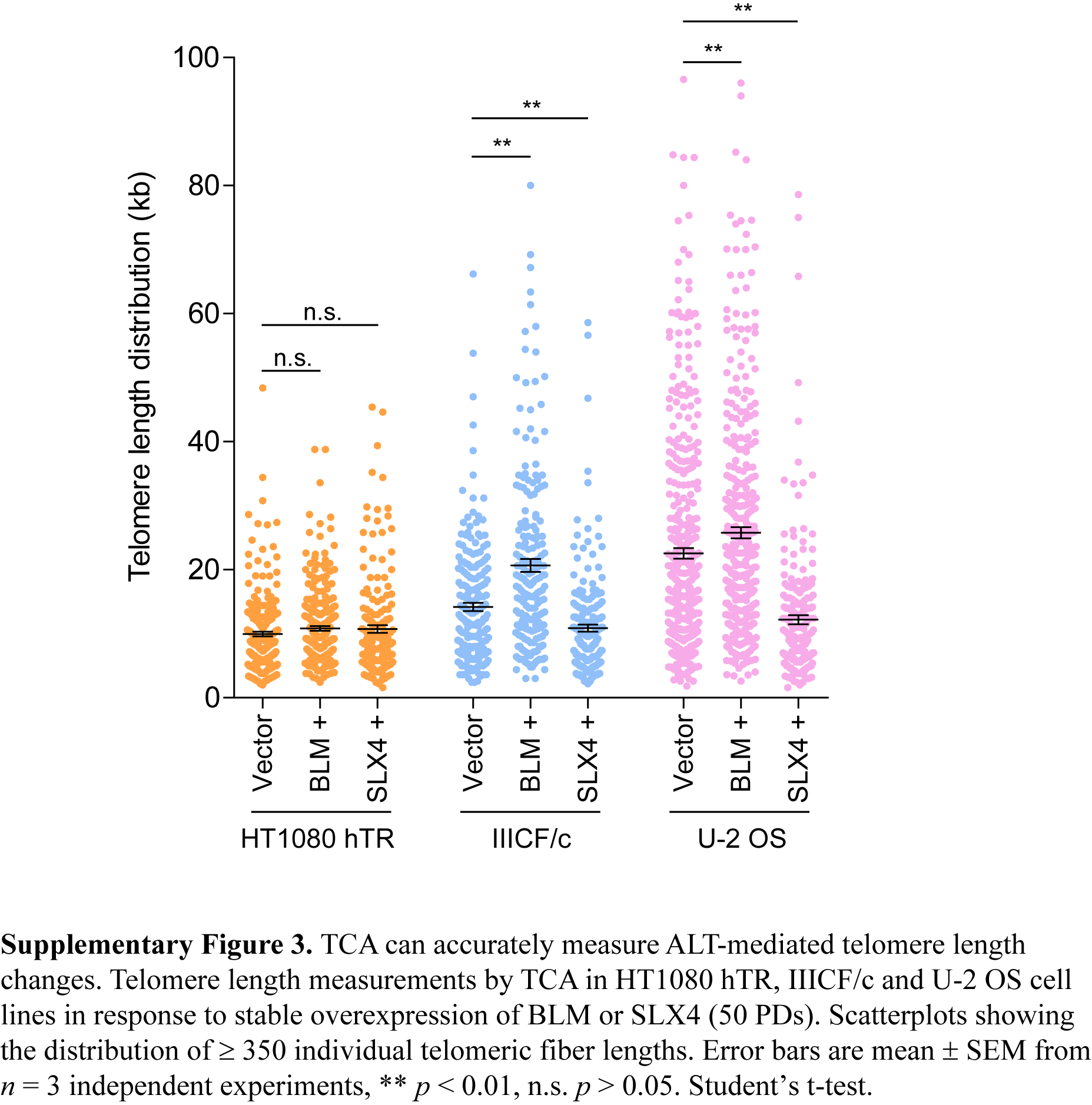

Supplement: Supplementary file 3 [file Image_3.tif]

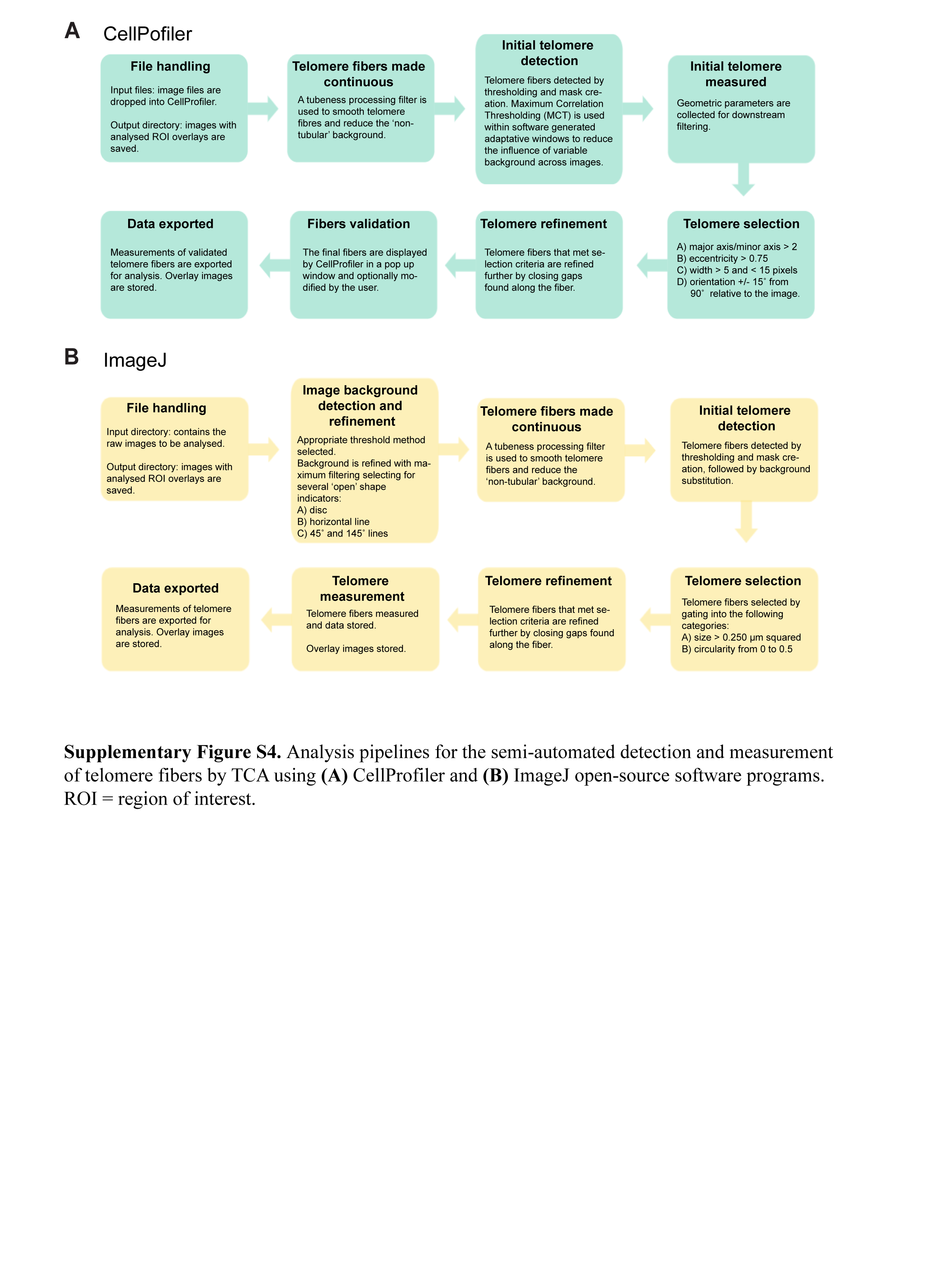

Supplement: Supplementary file 4 [file Image_4.tif]

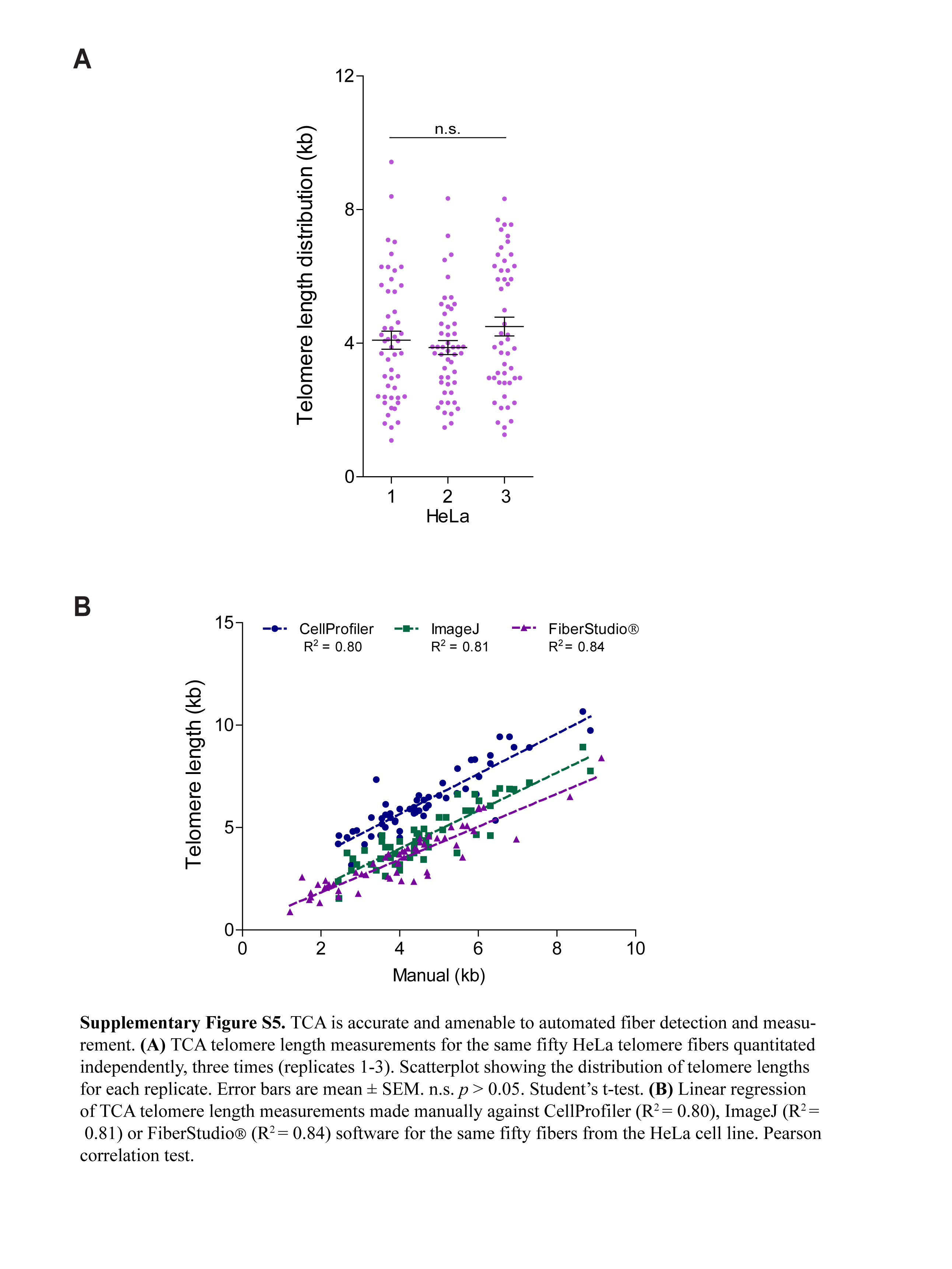

Supplement: Supplementary file 5 [file Image_5.tif]
