# Supplementary material for: Telomere Length Measurement by Molecular Combing
Source: Front Cell Dev Biol. 2020 Jun 16;8:493. doi: 10.3389/fcell.2020.00493 (PMC7308456; doi:10.3389/fcell.2020.00493)
Supplement: Supplementary file 6 [file Table_1.docx]

| Cell line | Cell type | Telomere maintenance mechanism | Mechanism of transformation | Ref |
| --- | --- | --- | --- | --- |
| MRC-5 | Human lung fibroblast | mortal | n/a | Jacobs et al., 1970 |
| HeLa | Human cervical adenocarcinoma | telomerase | tumour | Bryan et al., 1998; Gey et al., 1952 |
| HT1080 | Human connective tissue fibrosarcoma | telomerase | tumour | Rasheed et al., 1974 |
| HT1080 hTR | Human connective tissue fibrosarcoma | telomerase overexpression | tumour | Pickett et al., 2009 |
| IIICF/c | Human breast fibroblast from  an individual with Li–Fraumeni syndrome | ALT | spontaneous | Bryan et al., 1995 |
| U-2 OS | Human osteosarcoma | ALT | tumour | Heldin et al., 1986 |

**Supplementary Table S1.** Cell strain and derivative cell line information.

**References**

Bryan, T.M., Englezou, A., Dunham, M.A., and Reddel, R.R. (1998). Telomere length dynamics in telomerase-positive immortal human cell populations. Exp. Cell Res. 239, 370-378. doi: 10.1006/excr.1997.3907.

Bryan, T.M., Englezou, A., Gupta, J., Bacchetti, S., and Reddel, R.R. (1995). Telomere elongation in immortal human cells without detectable telomerase activity. EMBO J. 14, 4240-4248. doi: 10.1006/excr.1997.3907.

Gey, G.O., Coffman, W.D., and Kubicek, M. (1952). Tissue culture studies of the proliferative capacity of cervical carcinoma and normal epithelium. Cancer Res. 12, 264-265.

Heldin, C.H., Johnsson, A., Wennergren, S., Wernstedt, C., Betsholtz, C., and Westermark, B. (1986). A human osteosarcoma cell line secretes a growth factor structurally related to a homodimer of PDGF A-chains. Nature 319, 511-514. doi: 10.1038/319511a0.

Jacobs, J.P., Jones, C.M., and Baille, J.P. (1970). Characteristics of a human diploid cell designated MRC-5. Nature 227, 168-170. doi: 10.1038/227168a0.

Pickett, H.A., Cesare, A.J., Johnstone, R.L., Neumann, A.A., and Reddel, R.R. (2009). Control of telomere length by a trimming mechanism that involves generation of t-circles. EMBO J. 28, 799-809. doi: 10.1038/emboj.2009.42.

Rasheed, S., Nelson-Rees, W.A., Toth, E.M., Arnstein, P., and Gardner, M.B. (1974). Characterization of a newly derived human sarcoma cell line (HT-1080). Cancer 33, 1027-1033. doi: 10.1002/1097-0142(197404)33:4<1027::aid-cncr2820330419>3.0.co;2-z.
