# Supplementary material for: Telomere Length Measurement by Molecular Combing
Source: Front Cell Dev Biol. 2020 Jun 16;8:493. doi: 10.3389/fcell.2020.00493 (PMC7308456; doi:10.3389/fcell.2020.00493)
Supplement: Supplementary file 7 [file Data_Sheet_1.docx]

**Supplementary Material**

**TELOMERE LENGTH COMBING ASSAY (TCA): DETAILED PROTOCOL**

**REAGENTS**

Low melting point (LMP) agarose (Thermo Fisher Scientific)

0.5 M EDTA (pH 8.0)

10% (v/v) sarcosyl/0.5 M EDTA

20 mg/mL proteinase k (Sigma)

TE: 10 mM Tris-HCl,1 mM EDTA (pH 8.0)

MES: 0.5 M 2-(N-Morpholino)ethanesulfonic acid (pH 5.5) (Merck)

Agarase (Thermo Fisher Scientific)

YOYO-1^TM^ Iodide (Invitrogen)

ProLong™ gold antifade mountant (Thermo Fisher Scientific)

PBS

Ethanol (EtOH)

Telomeric PNA probe (TAMRA-OO-(CCCTAAA)_3_ or TAMRA-OO-KK(TTAGGG)_3_) (Panagene)

PNA hybridization buffer: 70% (v/v) deionized formamide, 0.25% (v/v) NEN blocking reagent (PerkinElmer), 10 mM Tris-HCl (pH 7.5), 4 mM Na_2_HPO_4_, 0.5 mM citric acid, 1.25 mM MgCl_2_

2x saline-sodium citrate (SSC)

ABDIL solution: 20 mM Tris (pH 7.5), 2% BSA, 0.2% fish gelatin, 150 mM NaCl, 0.1% Triton X-100, 0.1% sodium azide

**CONSUMABLES**

15 mL tubes

2 mL tubes with round bottom

Disposable plug molds (#1703713; BioRad)

Disposable DNA reservoirs (RES-001, Genomic Vision)

Engraved Combicoverslips (COV-002-RUO, Genomic Vision)

Microscope slides

**EQUIPMENT**

Water bath

Heat block

Vacuum pump

Tube rotator

FiberComb® Molecular Combing System (MCS-001, Genomic Vision)

Incubator

Shaker

Humidity chamber

Axio Imager.Z2 upright widefield microscope coupled to a Zeiss AxioCam 506 Mono camera (Carl Zeiss), or equivalent

**PROCEDURE**

**1.** Harvest cells to obtain 1x10^6^ cells in 45 µL PBS.

**2.** Pre-melt 2% LMP agarose at 68°C.

**3.** Mix equal volumes of cell solution and LMP agarose at 50°C in a water bath. Dispense 90 μL of cell and agarose solution per plug into disposable plug molds.

**4.** Allow plugs to set at 4ºC for 45 min.

PAUSE POINT: Store the plugs at 4ºC for up to 4 weeks. Do not freeze.

**5.** Sequentially aliquot 200 µL 0.5 M EDTA (pH 8.0), 25 µL of 10% (v/v) sarcosyl/0.5 M EDTA and 50 µL of 20 mg/mL proteinase k (Sigma) per plug into a 15 mL tube. Release plugs from the molds and immerse in the solution. Incubate the tubes at 50ºC for 12-16 h.

CRITICAL STEP: Ensure that the water bath is still, with non-turbulent water flow to minimize DNA shearing.

**6.** Discard the solution keeping the plug in the tube.

**7.** Add 13 mL TE and incubate at room temperature for 1 h on a tube rotator (slow speed). Discard the solution keeping the plug in the tube.

**8.** Repeat step 7 two more times.

PAUSE POINT: Store the plugs in EDTA at 4ºC for up to 4 weeks.

**9.** Perform a final wash with 13 mL TE incubating at room temperature for 3.5 h on a tube rotator.

**10.** Transfer the washed plug into a 2 mL tube containing 1 mL of MES, ensuring that the plug is immersed.

**11.** Incubate tubes on a heat block at 68ºC for 20 min, followed by 42ºC for 10 min.

**12.** Add 1.5 µL of agarase to each tube without removing from the heat block. Incubate at 42ºC for 12-16 h.

CRITICAL STEP: Before pipetting the agarase into the DNA solution, allow the agarase to warm up to room temperature for 10 s.

**13.** Add 1.2 mL of MES to a DNA reservoir.

**14.** Gently pour the DNA solution into the reservoir.

CRITICAL STEP: Do not micropipette as it will cause DNA shearing.

**15.** Fiber quality confirmation:

- Stretch DNA onto one coverslip per sample using the FiberComb® Molecular Combing System.
- Incubate coverslips at 60ºC for 1 h.
- Add 50 μL of YOYO-1^TM^ Iodide (diluted 2x10^-3^ in ABDIL) per microscope slide.
- Lower the coverslips onto the YOYO-1^TM^ Iodide with the engraved side facing down. Incubate at 37°C for 20 min.
- Wash coverslips with 2xSSC/0.1% Tween-20 at room temperature for 3 min on a shaker at medium speed. Repeat.
- Rinse coverslips with water.
- Air dry the coverslip at 37°C for 20 min and mount on microscope slides using ProLong™ Gold Antifade reagent.
- Confirm fiber quality and density at high magnification on an upright fluorescence widefield microscope. Shredded, tangled or sparse DNA fibers are considered low quality.

**16.** When fiber quality has been confirmed, stretch DNA fibers for all samples using the FiberComb® Molecular Combing System.

**17.** Incubate coverslips at 60ºC for 4 h to minimize photobreaking.

PAUSE POINT: Store the coverslips in the dark at room temperature for up to four weeks.

**18.** Allow coverslips to cool to room temperature for 5 min, followed by incubation with PBS at room temperature for 5 min.

**19.** Perform ethanol dehydration series on coverslips (70%, 90%, 100%).

**20.** Add 30 µL of telomeric PNA probe in PNA hybridization buffer (final concentration 0.3 μg/mL) per microscope slide.

**21.** Lower the coverslip onto the PNA probe solution with the engraved side facing down. Incubate at room temperature for 12-16 h in a humidity chamber in the dark.

**22.** Wash coverslips with pre-warmed 2xSSC at 60ºC for 10 min. *

**23.** Wash coverslips with 2xSSC/0.1% Tween-20 at room temperature for 5 min on a shaker at medium speed. *

**24.** Counterstain coverslips with YOYO-1^TM^ Iodide.

**25.** Rinse coverslips in water for 30 s and air dry.

**26.** Mount coverslips on microscope slides using ProLong™ Gold Antifade reagent.

**27.** Visualize fibers at high magnification on an upright widefield microscope coupled to a camera using the appropriate filters.

* More stringent washing steps were deemed too harsh and resulted in a diminished telomere signal.
